# Bi-allelic *ACBD6* variants lead to a neurodevelopmental syndrome with progressive and complex movement disorders

**DOI:** 10.1093/brain/awad380

**Published:** 2023-11-10

**Authors:** Rauan Kaiyrzhanov, Aboulfazl Rad, Sheng-Jia Lin, Aida Bertoli-Avella, Wouter W Kallemeijn, Annie Godwin, Maha S Zaki, Kevin Huang, Tracy Lau, Cassidy Petree, Stephanie Efthymiou, Ehsan Ghayoor Karimiani, Maja Hempel, Elizabeth A Normand, Sabine Rudnik-Schöneborn, Ulrich A Schatz, Marc P Baggelaar, Muhammad Ilyas, Tipu Sultan, Javeria Raza Alvi, Manizha Ganieva, Ben Fowler, Ruxandra Aanicai, Gulsen Akay Tayfun, Abdulaziz Al Saman, Abdulrahman Alswaid, Nafise Amiri, Nilufar Asilova, Vorasuk Shotelersuk, Patra Yeetong, Matloob Azam, Meisam Babaei, Gholamreza Bahrami Monajemi, Pouria Mohammadi, Saeed Samie, Selina Husna Banu, Jorge Pinto Basto, Fanny Kortüm, Mislen Bauer, Peter Bauer, Christian Beetz, Masoud Garshasbi, Awatif Hameed Issa, Wafaa Eyaid, Hind Ahmed, Narges Hashemi, Kazem Hassanpour, Isabella Herman, Sherozjon Ibrohimov, Ban A Abdul-Majeed, Maria Imdad, Maksudjon Isrofilov, Qassem Kaiyal, Suliman Khan, Brian Kirmse, Janet Koster, Charles Marques Lourenço, Tadahiro Mitani, Oana Moldovan, David Murphy, Maryam Najafi, Davut Pehlivan, Maria Eugenia Rocha, Vincenzo Salpietro, Miriam Schmidts, Adel Shalata, Mohammad Mahroum, Jawabreh Kassem Talbeya, Robert W Taylor, Dayana Vazquez, Annalisa Vetro, Hans R Waterham, Mashaya Zaman, Tina A Schrader, Wendy K Chung, Renzo Guerrini, James R Lupski, Joseph Gleeson, Mohnish Suri, Yalda Jamshidi, Kailash P Bhatia, Barbara Vona, Michael Schrader, Mariasavina Severino, Matthew Guille, Edward W Tate, Gaurav K Varshney, Henry Houlden, Reza Maroofian

**Affiliations:** Department of Neuromuscular Diseases, UCL Institute of Neurology, London WC1N 3BG, UK; Cellular and Molecular Research Center, Sabzevar University of Medical Sciences, Sabzevar 009851, Iran; Tübingen Hearing Research Centre, Department of Otolaryngology, Head and Neck Surgery, Eberhard Karls University, 72076 Tübingen, Germany; Genes & Human Disease Research Program, Oklahoma Medical Research Foundation, Oklahoma City, OK 73104, USA; Department of Medical Genetics, CENTOGENE GmbH, 18055 Rostock, Germany; Department of Chemistry, Imperial College London, Molecular Sciences Research Hub, London W12 0BZ, UK; Chemical Biology and Therapeutic Discovery Lab, The Francis Crick Institute, London NW1 1AT, UK; European Xenopus Resource Centre—XenMD, School of Biological Sciences, University of Portsmouth, Portsmouth PO1 2DT, UK; Clinical Genetics Department, Human Genetics and Genome Research Institute, National Research Centre, 12622 Cairo, Egypt; Genes & Human Disease Research Program, Oklahoma Medical Research Foundation, Oklahoma City, OK 73104, USA; Department of Neuromuscular Diseases, UCL Institute of Neurology, London WC1N 3BG, UK; Genes & Human Disease Research Program, Oklahoma Medical Research Foundation, Oklahoma City, OK 73104, USA; Department of Neuromuscular Diseases, UCL Institute of Neurology, London WC1N 3BG, UK; Genetics Research Centre, Molecular and Clinical Sciences Institute, St George’s University of London, London SW17 0RE, UK; Department of Medical Genetics, Next Generation Genetic Polyclinic, Mashhad 1696700, Iran; Institute of Human Genetics, University Medical Center Hamburg-Eppendorf, 20246 Hamburg, Germany; Institute of Human Genetics, University Hospital Heidelberg, Heidelberg 69120, Germany; Clinical Genomics Program, GeneDx, Gaithersburg, MD 20877, USA; Institute of Human Genetics, Medical University Innsbruck, Innsbruck 6020, Austria; Institute of Human Genetics, Medical University Innsbruck, Innsbruck 6020, Austria; Institute of Human Genetics, Technical University of Munich, Munich, 81675, Germany; Department of Chemistry, Imperial College London, Molecular Sciences Research Hub, London W12 0BZ, UK; Biomolecular Mass Spectrometry & Proteomics Group, Utrecht University, 3584 CH Utrecht, The Netherlands; Department of BioEngineering, University of Engineering and Applied Sciences, 19130 Swat, Pakistan; Centre for Omic Sciences, Islamia College University, 25000 Peshawar, Pakistan; Department of Pediatric Neurology, Institute of Child Health, Children Hospital, Lahore 54600, Pakistan; Department of Pediatric Neurology, Institute of Child Health, Children Hospital, Lahore 54600, Pakistan; Department of Neurology, Avicenna Tajik State Medical University, 734063 Dushanbe, Tajikistan; Imaging Core, Oklahoma Medical Research Foundation, Oklahoma City, OK 73104, USA; Department of Medical Genetics, CENTOGENE GmbH, 18055 Rostock, Germany; Department of Pediatric Genetics, Marmara University Medical School, 34722 Istanbul, Turkey; Pediatric Neurology Department, National Neuroscience Institute, King Fahad Medical City, 49046 Riyadh, Saudi Arabia; King Saud Bin Abdulaziz University for Health Sciences, Department of Pediatrics, King Abdullah Specialized Children’s Hospital, Riyadh 11461, Saudi Arabia; International Collaboration on Repair Discoveries (ICORD), University of British Columbia, Vancouver, BC V5Z 1M9, Canada; Department of Neurology, Avicenna Tajik State Medical University, 734063 Dushanbe, Tajikistan; Center of Excellence for Medical Genomics, Department of Pediatrics, King Chulalongkorn Memorial Hospital, Faculty of Medicine, Chulalongkorn University, Bangkok 10330, Thailand; Division of Human Genetics, Department of Botany, Faculty of Science, Chulalongkorn University, Bangkok 10330, Thailand; Pediatrics and Child Neurology, Wah Medical College, 47000 Wah Cantt, Pakistan; Department of Pediatrics, North Khorasan University of Medical Sciences, Bojnurd 94149-74877, Iran; Pars Advanced and Minimally Invasive Medical Manners Research Center, Pars Hospital, Tehran, Iran; Children’s Medical Center, Pediatrics Center of Excellence, Ataxia Clinic, Tehran University of Medical Sciences, Tehran 1416634793, Iran; Faculty of Medical Sciences, Department of Medical Genetics, Tarbiat Modares University, Tehran 1411944961, Iran; Pars Advanced and Minimally Invasive Medical Manners Research Center, Pars Hospital, Tehran, Iran; Department of Paediatric Neurology and Development, Dr. M.R. Khan Shishu (Children) Hospital and Institute of Child Health, Dhaka 1216, Bangladesh; Department of Medical Genetics, CENTOGENE GmbH, 18055 Rostock, Germany; Institute of Human Genetics, University Medical Center Hamburg-Eppendorf, 20246 Hamburg, Germany; Division of Clinical Genetics and Metabolism, Nicklas Children's Hospital, Miami, FL 33155, USA; Department of Medical Genetics, CENTOGENE GmbH, 18055 Rostock, Germany; Department of Medical Genetics, CENTOGENE GmbH, 18055 Rostock, Germany; Faculty of Medical Sciences, Department of Medical Genetics, Tarbiat Modares University, Tehran 1411944961, Iran; Department of Neurology, University of Basrah, 61004 Basrah, Iraq; Department of Genetics and Precision Medicine, King Abdullah International Medical Research Centre, King Saud bin Abdulaziz University for Health Science, King Abdulaziz Medical City, Ministry of National Guard-Health Affairs (NGHA), Riyadh 11426, Saudi Arabia; Department of Genetics and Precision Medicine, King Abdullah International Medical Research Centre, King Saud bin Abdulaziz University for Health Science, King Abdulaziz Medical City, Ministry of National Guard-Health Affairs (NGHA), Riyadh 11426, Saudi Arabia; Department of Pediatrics, School of Medicine, Mashhad University of Medical Sciences, 13131–99137 Mashhad, Iran; Non-Communicable Diseases Research Center, Sabzevar University of Medical Sciences, 319 Sabzevar, Iran; Section of Pediatric Neurology and Developmental Neuroscience, Department of Pediatrics, Baylor College of Medicine, Houston, TX 68010, USA; Department of Molecular and Human Genetics, Baylor College of Medicine, Houston, TX 77030, USA; Department of Neurology, Texas Children’s Hospital, Houston, TX 77030, USA; Pediatric Neurology, Neurogenetics and Rare Diseases, Boys Town National Research Hospital, Boys Town, NE 68131, USA; Department of Neurology, Avicenna Tajik State Medical University, 734063 Dushanbe, Tajikistan; Molecular Pathology and Genetics, The Pioneer Molecular Pathology Lab, Baghdad 10044, Iraq; Centre for Human Genetics, Hazara University, 21300 Mansehra, Pakistan; Department of Neurology, Avicenna Tajik State Medical University, 734063 Dushanbe, Tajikistan; Department of Pediatric Neurology, Clalit Health Care, 2510500 Haifa, Israel; Department of Medical Genetics, CENTOGENE GmbH, 18055 Rostock, Germany; SOM-Peds-Genetics, University of Mississippi Medical Center, Jackson MS, 39216, USA; Laboratory Genetic Metabolic Diseases, Amsterdam University Medical Centers location AMC, 1100 DD Amsterdam, The Netherlands; Faculdade de Medicina, Centro Universitario Estácio de Ribeirão Preto, 14096-160 São Paulo, Brazil; Department of Molecular and Human Genetics, Baylor College of Medicine, Houston, TX 77030, USA; Serviço de Genética Médica, Departamento de Pediatria, Hospital de Santa Maria, Centro Hospitalar Universitário de Lisboa Norte, 1649-035 Lisboa, Portugal; Department of Clinical and Movement Neurosciences, UCL Queen Square Institute of Neurology, University College London, London WC1N 3BG, UK; Pediatrics Genetics Division, Center for Pediatrics and Adolescent Medicine, Faculty of Medicine, Freiburg University, 79106 Freiburg, Germany; Genome Research Division, Human Genetics Department, Radboud University Medical Center, 6500 HB Nijmegen, The Netherlands; Section of Pediatric Neurology and Developmental Neuroscience, Department of Pediatrics, Baylor College of Medicine, Houston, TX 68010, USA; Department of Molecular and Human Genetics, Baylor College of Medicine, Houston, TX 77030, USA; Department of Medical Genetics, CENTOGENE GmbH, 18055 Rostock, Germany; Department of Neuromuscular Diseases, UCL Institute of Neurology, London WC1N 3BG, UK; Pediatrics Genetics Division, Center for Pediatrics and Adolescent Medicine, Faculty of Medicine, Freiburg University, 79106 Freiburg, Germany; Genome Research Division, Human Genetics Department, Radboud University Medical Center, 6500 HB Nijmegen, The Netherlands; CIBSS-Centre for Integrative Biological Signalling Studies, University of Freiburg, Freiburg, Germany; Pediatrics and Medical Genetics, the Simon Winter Institute for Human Genetics, Bnai Zion Medical Center, 31048 Haifa, Israel; Bruce Rappaport Faculty of Medicine, the Technion institution of Technology, 3200003 Haifa, Israel; CIBSS-Centre for Integrative Biological Signalling Studies, University of Freiburg, Freiburg, Germany; Pediatrics and Medical Genetics, the Simon Winter Institute for Human Genetics, Bnai Zion Medical Center, 31048 Haifa, Israel; Department of Radiology, The Bnai Zion Medical Center, Haifa 31048, Israel; Wellcome Centre for Mitochondrial Research, Translational and Clinical Research Institute, Faculty of Medical Sciences, Newcastle University, Newcastle upon Tyne NE2 4HH, UK; NHS Highly Specialised Service for Rare Mitochondrial Disorders, Newcastle upon Tyne Hospitals NHS Foundation Trust, Newcastle upon Tyne NE1 4LP, UK; Division of Clinical Genetics and Metabolism, Nicklas Children's Hospital, Miami, FL 33155, USA; Neuroscience Department, Meyer Children's Hospital IRCCS, 50139 Florence, Italy; Laboratory Genetic Metabolic Diseases, Amsterdam University Medical Centers location AMC, 1100 DD Amsterdam, The Netherlands; Department of Paediatric Neurology and Development, Dr. M.R. Khan Shishu (Children) Hospital and Institute of Child Health, Dhaka 1216, Bangladesh; Department of Biosciences, University of Exeter, Exeter EX4 4QD, UK; Department of Pediatrics, Columbia University Irving Medical Center, New York, NY 10032, USA; Department of Medicine, Columbia University Irving Medical Center, New York, NY 10032, USA; Neuroscience Department, Meyer Children's Hospital IRCCS, 50139 Florence, Italy; Neuroscience, Pharmacology and Child Health Department, University of Florence, 50139 Florence, Italy; Department of Molecular and Human Genetics, Baylor College of Medicine, Houston, TX 77030, USA; Department of Neurology, Texas Children’s Hospital, Houston, TX 77030, USA; Human Genome Sequencing Center, Baylor College of Medicine, Houston, TX 77030, USA; Department of Neurosciences, University of California, San Diego, CA 92093, USA; Department of Neurosciences, Rady Children's Institute for Genomic Medicine, San Diego, CA 92025, USA; Clinical Genetics Service, Nottingham University Hospitals NHS Trust, Nottingham NG5 1PB, UK; Genetics Research Centre, Molecular and Clinical Sciences Institute, St George’s University of London, London SW17 0RE, UK; Human Genetics Centre of Excellence, Novo Nordisk Research Centre Oxford, Oxford, OX3 7FZ, UK; Department of Clinical and Movement Neurosciences, UCL Queen Square Institute of Neurology, University College London, London WC1N 3BG, UK; Tübingen Hearing Research Centre, Department of Otolaryngology, Head and Neck Surgery, Eberhard Karls University, 72076 Tübingen, Germany; Institute of Human Genetics, University Medical Center Göttingen, 37073 Göttingen, Germany; Institute for Auditory Neuroscience and Inner Ear Lab, University Medical Center Göttingen, 37075 Göttingen, Germany; Department of Biosciences, University of Exeter, Exeter EX4 4QD, UK; Neuroradiology Unit, IRCCS Istituto Giannina Gaslini, 16147 Genoa, Italy; European Xenopus Resource Centre—XenMD, School of Biological Sciences, University of Portsmouth, Portsmouth PO1 2DT, UK; Department of Chemistry, Imperial College London, Molecular Sciences Research Hub, London W12 0BZ, UK; Chemical Biology and Therapeutic Discovery Lab, The Francis Crick Institute, London NW1 1AT, UK; Genes & Human Disease Research Program, Oklahoma Medical Research Foundation, Oklahoma City, OK 73104, USA; Department of Neuromuscular Diseases, UCL Institute of Neurology, London WC1N 3BG, UK; Department of Neuromuscular Diseases, UCL Institute of Neurology, London WC1N 3BG, UK

**Keywords:** *ACBD6*, neurodegeneration, dystonia, ataxia, parkinsonism, *N*-myristoylation

## Abstract

The acyl-CoA-binding domain-containing protein 6 (ACBD6) is ubiquitously expressed, plays a role in the acylation of lipids and proteins and regulates the *N*-myristoylation of proteins via *N*-myristoyltransferase enzymes (NMTs). However, its precise function in cells is still unclear, as is the consequence of *ACBD6* defects on human pathophysiology. Using exome sequencing and extensive international data sharing efforts, we identified 45 affected individuals from 28 unrelated families (consanguinity 93%) with bi-allelic pathogenic, predominantly loss-of-function (18/20) variants in *ACBD6*. We generated zebrafish and *Xenopus tropicalis acbd6* knockouts by CRISPR/Cas9 and characterized the role of *ACBD6* on protein *N*-myristoylation with myristic acid alkyne (YnMyr) chemical proteomics in the model organisms and human cells, with the latter also being subjected further to ACBD6 peroxisomal localization studies. The affected individuals (23 males and 22 females), aged 1–50 years, typically present with a complex and progressive disease involving moderate-to-severe global developmental delay/intellectual disability (100%) with significant expressive language impairment (98%), movement disorders (97%), facial dysmorphism (95%) and mild cerebellar ataxia (85%) associated with gait impairment (94%), limb spasticity/hypertonia (76%), oculomotor (71%) and behavioural abnormalities (65%), overweight (59%), microcephaly (39%) and epilepsy (33%). The most conspicuous and common movement disorder was dystonia (94%), frequently leading to early-onset progressive postural deformities (97%), limb dystonia (55%) and cervical dystonia (31%). A jerky tremor in the upper limbs (63%), a mild head tremor (59%), parkinsonism/hypokinesia developing with advancing age (32%) and simple motor and vocal tics were among other frequent movement disorders. Midline brain malformations including corpus callosum abnormalities (70%), hypoplasia/agenesis of the anterior commissure (66%), short midbrain and small inferior cerebellar vermis (38% each) as well as hypertrophy of the clava (24%) were common neuroimaging findings. *Acbd6*-deficient zebrafish and *Xenopus* models effectively recapitulated many clinical phenotypes reported in patients including movement disorders, progressive neuromotor impairment, seizures, microcephaly, craniofacial dysmorphism and midbrain defects accompanied by developmental delay with increased mortality over time. Unlike ACBD5, ACBD6 did not show a peroxisomal localization and ACBD6-deficiency was not associated with altered peroxisomal parameters in patient fibroblasts. Significant differences in YnMyr-labelling were observed for 68 co- and 18 post-translationally *N*-myristoylated proteins in patient-derived fibroblasts. *N*-myristoylation was similarly affected in *acbd6*-deficient zebrafish and *X. tropicalis* models, including Fus, Marcks and Chchd-related proteins implicated in neurological diseases. The present study provides evidence that bi-allelic pathogenic variants in *ACBD6* lead to a distinct neurodevelopmental syndrome accompanied by complex and progressive cognitive and movement disorders.

## Teaser

Kaiyrzhanov *et al.* report that mutations in the *ACBD6* gene cause a distinct neurodevelopmental syndrome accompanied by complex and progressive cognitive and movement disorders in a cohort of 45 patients from 28 families. Zebrafish and frog models and proteomics studies shed light on the disease mechanism.

## Introduction

Acyl-CoA-binding domain-containing proteins (ACBDs) are a large multigene family of intracellular lipid-binding proteins, and in mammals, they include ACBD1–7. These proteins specifically bind long-chain fatty acyl-CoA esters (LCACoA) and control their intracellular concentration.^1,2^ Various cellular functions have been ascribed to this protein family, ranging from lipid homeostasis, organelle formation, apoptotic responses and intracellular vesicle trafficking as well as tethering between the peroxisome and endoplasmic reticulum.^2^ ACBDs have been suggested to play a crucial role in brain development via their strong proliferative effects on the neural stem and progenitor cells.^3^ To date, defects in only two ACBD members have been associated with Mendelian disorders in humans. *ACBD5* deficiency has been reported to cause a combination of retinal dystrophy with leukodystrophy and defects in peroxisomal very long-chain fatty acid metabolism in four families.^4-7^*ACBD6* has been suggested as a candidate gene for intellectual disability (ID) in two large-cohort gene discovery studies reporting limited phenotypic data^8,9^ and in a case report describing two siblings with neurodevelopmental disorder, obesity, pancytopenia, diabetes mellitus, cirrhosis and renal failure but with a limited neurological phenotype.^10^

The ACBD6 protein has two domains: the N-terminal Acyl-CoA-binding (ACB) and the specialized C-terminal ankyrin repeat (ANK) domain. This protein is detected in the cytosol and nuclei of cells and modulates the acylation of lipids and proteins.^11^ It has also been suggested that ACBD6 is associated with *N*-myristoyltransferases (NMTs) in human cells by ligand binding and protein interaction, although direct evidence for this in cells is lacking.^12^ In humans, NMT1 and NMT2 enzymatically catalyse the *N*-myristoylation of substrate proteins by transferring the myristate from myristoyl coenzyme A (Myr-CoA) onto the N-terminal glycine of nascent proteins (co-translationally, at the ribosome) or to internal glycines uncovered by protease cleavages during apoptosis (post-translationally).^13^ The global *N*-myristoylated proteome consists of more than 200 co- and post-translationally *N*-myristoylated proteins in humans,^14,15^ and *N*-myristoylation is important for the association of substrates with membranes and their interaction with other proteins.^16^

Here, we describe 45 affected individuals from 28 unrelated families with 18 bi-allelic predicted loss-of-function (LOF), 1 missense and 1 in-frame insertion variants in *ACBD6* presenting a new and distinct neurodevelopmental syndrome with a complex and progressive movement disorder phenotype. We performed functional studies in zebrafish and *Xenopus tropicalis* knockouts generated by clustered regularly interspaced short palindromic repeats (CRISPR)/Cas9 that recapitulate many clinical features reported in patients. We ruled out a major role for *ACBD6* in peroxisomes and investigated the deregulation of co- and post-translationally *N*-myristoylated proteins in *ACBD6* deficient human, zebrafish and *X. tropicalis* cells, given its putative role in modulating NMT activity.

## Materials and methods

### Patient identification and deep phenotyping

Using the GeneMatcher platform,^17^ extensive international data sharing and screening the variant databases of several research and diagnostic laboratories worldwide, we identified 27 families with bi-allelic variants in *ACBD6*. Follow-up details were obtained in the family reported by Yeetong *et al*.^10^ For a comprehensive phenotypic characterization of the affected individuals, we obtained clinical details, including neurological examination, via a universally-adopted proforma (Supplementary Table 1). Where possible, video recordings and facial photographs of the affected individuals along with their brain MRIs were made available for review by a movement disorders specialist (K.B.), dysmorphologist (M.S.) and neuroradiologist (M.Sev.), respectively. Parents and legal guardians of all affected individuals consented to the publication of clinical and genetic information, including video and photographs, according to the Declaration of Helsinki, and the respective local Ethics Committees approved the study.

### Genetic analysis

Using genomic DNA from whole blood samples of the probands from the 28 unrelated families, whole-exome sequencing (WES) was performed at 10 different centres worldwide using methods specified in Supplementary Table 1. WES data analysis and variant filtering and prioritization were performed using in-house implemented pipelines at different centres (Supplementary Table 1). Genotyping and homozygosity mapping were done in Families 1, 3, 5, 7 and 21 according to standard procedures using the Automap software (https://automap.iob.ch/). Sanger sequencing was performed to confirm co-segregation in all available family members. mRNA expression analysis for *ACBD6* was performed by relative real-time PCR (Supplementary material, ‘Method 1’ section). RNA studies to assay the effect of the splice acceptor site variants were performed as previously described^18,19^ (Supplementary material, ‘Method 2’ section).

### Functional studies in zebrafish

Zebrafish (*Danio rerio*) were raised and maintained under standard conditions in an Association for Assessment and Accreditation of Laboratory Animal Care (AALAC)-accredited facility at the Oklahoma Medical Research Foundation (OMRF). All experiments were performed as per protocol 22-18 approved by the Institutional Animal Care Committee of OMRF (IACUC). Wild-type zebrafish strain (*NHGRI-1*)^20^ or transgenic lines as described were used for all experiments. Detailed experimental procedures are described in the Supplementary material, ‘Method 3’ section.

### Functional studies in *Xenopus tropicalis*

*X. tropicalis* was used to test the gene-disease link for *ACBD6* further in a second animal model. The details of *X. tropicalis* care, generating F0 *acbd6* crispant animals, and phenotypic analysis of F0 *acbd6* crispant animals are provided in the Supplementary material, ‘Method 4’ section. All procedures were conducted in accordance with the Home Office Code of Practice under PP4353452, with approval from the University of Portsmouth’s Animal Welfare and Ethical Review Body.

### Cell culture

Human fibroblasts were obtained from skin biopsies of two unrelated affected individuals, F1:S1 and F2:S1, with a single base change in *ACBD6* affecting the splice acceptor site in intron 5 and a frameshift variant c.82dupG; p.(Val28GlyfsTer6), respectively. A control cell line from the unaffected sibling from F1 (a homozygous wild-type sibling) was established (control 1). Wild-type human control (C109) skin fibroblasts were provided by H. Waterham (control 2). COS-7 cells were cultured to perform immunofluorescence and microscopy analysis of ACBD6 localization (Supplementary material, ‘Method 5’ section).

### Analysis of peroxisomal chain fatty acid β-oxidation parameters

A D3-C22:0 loading test was performed by loading cells for 3 days with 30 µM deuterated (D3) C22:0 followed by fatty acid analysis with tandem mass spectrometry, essentially as previously described.^21^

### Myristic acid alkyne chemical proteomics, whole proteome analysis and meta-analyses

Wild-type and ACBD6 deficient human fibroblasts, zebrafish and *X. tropicalis* embryos were metabolically labelled in the presence or absence of myristic acid alkyne (YnMyr), followed by unbiased whole proteome analysis or chemical proteomics after YnMyr-enrichment to identify and quantify proteins *N*-myristoylated with YnMyr. Sample preparation, processing and data analyses were performed as described previously, including the calculation of false discovery rate (FDR) adjusted *P*-values.^22,23^ Meta-analysis for pathway- and disease-enrichment analyses were performed in Metacore (Clarivate). Detailed experimental procedures are described in the Supplementary material, ‘Method 6’ section.

### *Acbd6* expression studies in mouse brain

Gene expression in the adult mouse brain was performed and visualized as described in the Supplementary material, ‘Method 7’ section.

## Results

**Figure 1:**
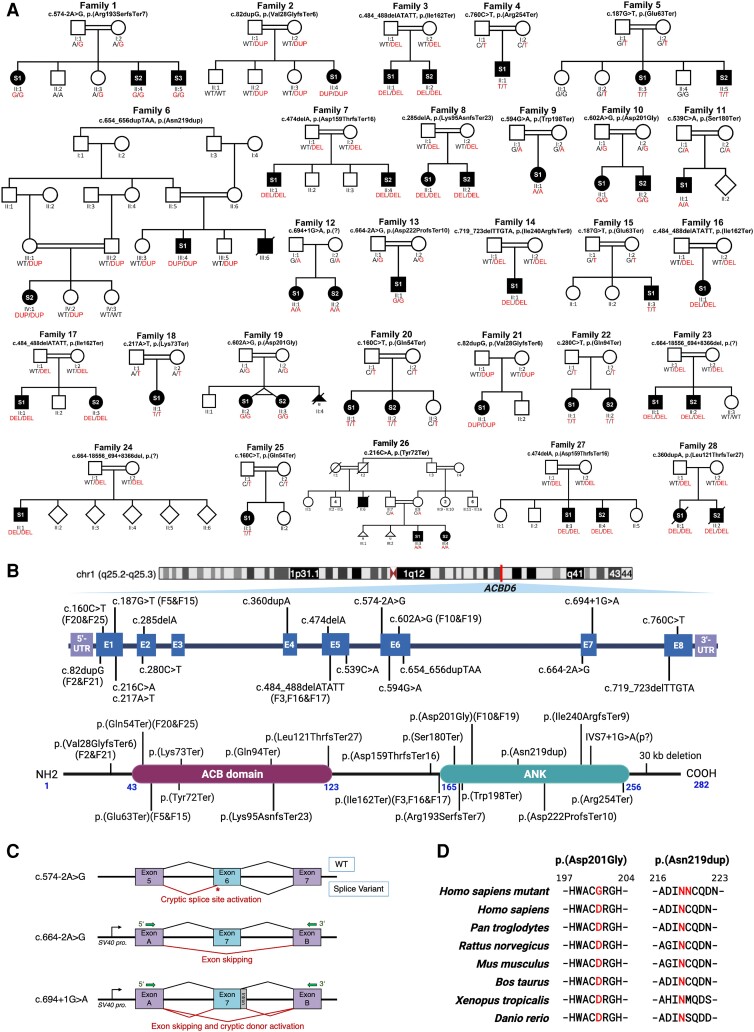
**Family pedigrees, schematic variants’ representation, conserved regions of substitution variants in *ACBD6,* and splicing effects**. (**A**) Pedigrees and segregation results for the 28 unrelated families. Double lines between individuals represent consanguinity. The 45 affected individuals recruited for the study are shaded and indicated with their respective subject (S) number (S1, S2 or S3). The segregation data for all individuals tested via Sanger sequencing are shown with the presence of the *ACBD6* variant (red) and/or the reference allele (black), two red/black texts indicate a homozygous state and one red + one black text indicates a heterozygous state. The genotyping is based on the coding DNA sequence. DEL = deletion; DUP = duplication; WT = wild-type. (**B**) *Top*: Schematic representation of the gene and protein positions of detected variants in *ACBD6*. *ACBD6* is located on chromosome 1 at cytogenetic position q25.2q25.3. *Middle*: The genetic variants mapped to the NM_032360.4 transcript of *ACBD6*. *Bottom*: *ACBD6* variants mapped on the protein level. Three variants, including p.(Gly22fs), p.(Leu121ThrfsTer27) and a 30 kb deletion in the C-terminus, have been reported previously.^6,7,10^ Recurrent variants are labelled with family codes. (**C**) Splicing schematic for the c.574-2A>G (*top*) variant in *ACBD6* showing cryptic acceptor splice site activation in exon 6. The c.664-2A>G (*middle*) and c.694+1G>A (*bottom*) variants affect splicing of exon 7 both show exon skipping. Additionally, the c.694+1G>A variant activates a cryptic donor splice site. (**D**) Interspecies alignment performed with Clustal Omega showing the complete conservation down to invertebrates of the amino acid residues affected by a missense variant leading to an amino acid substitution p.(Asp201Gly) and an in-frame duplication p.(Asn219dup).

Using WES and homozygosity mapping, we identified 20 homozygous variants in *ACBD6* across 28 unrelated families. Variants were subjected to familial segregation testing (Fig. 1A) and assessed using *in silico* analysis tools and genomic databases (Table 1 and Supplementary Tables 1 and 2). All variants are listed using the canonical transcript NM_032360.4 (Fig. 1B). Family 1 showed a splice variant (c.574-2A>G), which has been shown to result in cryptic splice activation after five nucleotides in exon 6 [r.574_578del, p.(Arg193SerfsTer7)] (Fig. 1C and Supplementary Fig. 1). Families 12 (c.694+1G>A) and 13 (c.664-2A>G) each harboured novel splice variants that resulted in skipping of exon 7, leading to a frameshift and premature termination [r.664_694del, p.(Asp222ProfsTer10)]. The c.694+1G>A variant showed additional evidence of activation of a cryptic splice donor site, also causing a frameshift and premature termination [r.694_694+1ins23, p.(Ala232AspfsTer8)] (Fig. 1C and Supplementary Fig. 2). Families 7 and 27, 8, 14 and 28 carried homozygous frameshift variants including c.474delA, p.(Asp159ThrfsTer16) (Families 7 and 27 shared this *ACBD6* variant); c.285delA, p.(Lys95AsnfsTer23); c.719_723delTTGTA, p.(Ile240ArgfsTer9); and c.360dupA, p.(Leu121ThrfsTer27), respectively. Families 2 and 21 shared the same homozygous frameshift *ACBD6* c.82dupG, p.(Val28GlyfsTer6) variant, as was the case with Families 3, 16 and 17, who harboured the same homozygous frameshift *ACBD6* c.484_488delATATT, p.(Ile162Ter) variant. Families 4, 9, 11, 18, 20, 22 and 26 carried homozygous nonsense variants, including: c.760C>T, p.(Arg254Ter); c.594G>A, p.(Trp198Ter); c.539C>A, p.(Ser180Ter); c.217A>T, p.(Lys73Ter); c.160C>T, p.(Gln54Ter); c.280 C>T, p.(Gln94Ter); and c.216C>A, p.(Tyr72Ter), respectively. Families 5 and 15 shared the same homozygous nonsense *ACBD6* c.187G>T, p.(Glu63Ter) variant. Likewise, Families 20 and 25 shared the same homozygous nonsense *ACBD6* c.160C>T, p.(Gln54Ter) variant. Families 23 and 24 each harboured a large deletion variant [c.664-18556_694+8366del, p.(?)], which spanned 26 953 bp of the *ACBD6* sequence and included the complete deletion of exon 7. The affected individuals in Family 6 carried an in-frame duplication variant c.654_656dupTAA, p.(Asn219dup) in exon 6. Families 10 and 19 harboured the same homozygous predicted-deleterious missense *ACBD6* variant c.602A>G, p.(Asp201Gly) in exon 6 (Fig. 1D). All variants were ultra-rare or absent in ∼1.8 million alleles inspected through a number of large genetic variant databases listed in Supplementary Table 2. A detailed description of the variants is provided in Supplementary Tables 1 and 2.

**Table 1:** Main clinical features of affected individuals with homozygous *ACBD6* variants

Family ID	1	2	3	4	5	6	7	8	9	10	11	12	13	14	15	16	17	18	19	20	21	22	23	24	25	26	27	28
Affected, *n*	3	1	2	1	2	2	2	2	1	2	1	2	1	1	1	1	2	1	2	2	1	2	2	1	1	2	2	2
GDD/ID	3+	–	2+	+	2+	2+	2+	2+	+	2+	+	2+	+	+	+	+	2+	+	2+	2+	+	2+	2+	+	+	2+	2+	2+
Progressive disease course	3+	X	2+	X	2+	2+	2+	2+	X	2+	+	2X	+	+	+	X	2+	X	2X	2X	+	2X	2+	+	X	2X	2+	2+
Microcephaly	1+	−	2−	+	2−	1+	1+	2+	X	2−	−	2−	+	−	−	+	2X	−	2+	2X	−	2X	2−	+	X	2X	1+	2X
Short stature	3−	+	1+	X	2−	2+	2+	2+	−	2−	+	2−	+	+	−	X	2X	X	2−	2+	+	2X	1−, 1X	+	X	2X	2X	2X
Facial dysmorphism	3+	+	2+	X	2+	2+	2+	2+	+	2+	+	2+	+	+	+	+	2+	X	2+	2+	+	2X	2−	+	X	2X	2+	2+
Oculomotor abnormalities	3X	X	2+		2+	2+	2X	1+	X	2+	−	2+	+	−	+	+	2X	+	2−	1+	+	2X	2−	X	X	2X	2+	2X
Cerebellar ataxia	3+	+	1+	+	2+	2+	2+	2+	−	2+	+	2+	+	+	+	+	2+	X	2+	2+	−	2X	2−	−	X	2+	2+	2+
Limb spasticity/hypertonia	3+	+	2+	+	2+	1+	2+	2+	−	2+	−	2−	+/−	+	+/−	+	2+	X	2+	2−	−	2X	1+, 1−	−	X	2+	2+	2+
Gait abnormalities	1+	+	2+	+	2+	2+	2+	2+	−	2+	+	2+	+	X	+	+	2+	+	2+	2X	X	2X	1+, 1X	X	X	2+	2+	2+
No independent gait	1+	+	2−	X	2−	2−	2−	2−	−	1+	−	2−	−	+	−	−	1+	−	2+	2+	+	2X	1−, 1X	+	+	2X	2−	2−
Hypokinesia/parkinsonism	3+	X	1+	X	2−	2+	2X	2−	X	2X	X	2−	−	−	−	+	1+	X	2−	2−	−	2X	2−	−	X	2X	1+	1+
Truncal/limb dystonia	3+	X	2+	X	2+	2+	2X	2+	X	2+	+	1+, 1−	+	+	+	+	2+	X	2+	2+	+	2X	2X	−	X	2X	2+	2+
Upper limb/head tremor	3X	+	2+	+	2+	2+	2−	2−	−	2+	+	2−	+	+	+	+	2+	+	2+	2−	−	2X	2−	−	X	2X	2+	2X
Tics and TLV	1+	X	1+	X	2X	2X	2X	2+	X	2−	−	2X	+	X	X	X	2X	−	2X	1+	−	2X	2−	−	X	2X	1+	2X
Postural instability	2+	X	2−	X	2+	2X	2X	2+	X	2+	+	2−	+	+	+	+	2X	X	2X	2X	X	2X	1−, 1X	X	X	2X	2+	2+
Epileptic seizures	2+	+	2−	+	2+	2−	2−	2−	+	2−	−	2−	+	−	−	+	2X	−	2−	2−	+	2X	1+, 1−	−	+	2X	2−	2−
Behavioural problem	3+	−	2−	−	2+	2+	2+	2−	−	2+	−	2−	+	+	+	+	2X	X	2+	2+	−	2X	1+, 1−	−	X	2X	2+	2+
Premature ageing	3+	−	2−	−	2−	1+	1+	2−	−	2−	−	2−	−	−	−	−	2+	−	2−	2−	−	2X	2−	−	X	2X	2−	2+
Reduction of PWM	2+^a^	X	1+	X	1+	1+	1+	1−^a^	X	2+	−	2X	+	−	−	X	2X	X	2−	2−	X	2X	2X	X	X	2X	2−	2X
CC hypoplasia/agenesis	2+^a^	+	1+	X	1+	1+	2+	1−^a^	X	2+	+	2X	−	+	−	+	2+	X	2+	2−	+	2X	2X	X	X	2X	2+	2X
AC hypoplasia/agenesis	2+^a^	X	2−	X	2+	2+	2+	1+^a^	X	2+	+	2X	+	+	+	X	2X	X	2+	2−	X	2X	2X	X	X	2X	2+	2X
Short midbrain	2+^a^	X	2−	X	1+	2+	1+	1+^a^	X	2+	+	2X	−	+	−	X	2X	X	2−	2−	X	X	2X	X	X	2X	2−	2X
ICV hypoplasia	2+^a^	X	2−	X	1+	2+	1+	1+^a^	X	1+	−	2X	+	+	−	X	2X	X	2−	2−	+	2X	2X	X	X	2X	2−	2X
Hypertrophy of the clava	2−^a^	X	2−	X	2−	1+	1−	1−^a^	X	2−	−	2X	+	+	−	X	2X	X	2+	2−	X	2X	2X	X	X	2X	2−	2X

− = negative for the feature of interest; + = positive for the feature of interest; +/− = mild hypertonia; AC = anterior commissure; CC = corpus callosum; F = family; GDD = global developmental delay; ICV = hypoplasia of the inferior cerebellar vermis; ID = intellectual disability; PWM = periventricular white matter; TLV = tic-like vocalizations; X = not available/not applicable. The numbers preceding the symbols ‘+’, ‘−’ and ‘X’ indicate the number of siblings who are positive, negative or have no data on the feature of interest in families with multiple affected individuals.

^a^Brain MRI scans are not available from another affected sibling.

### Clinical delineation of *ACBD6-*related disease

**Figure 2:**
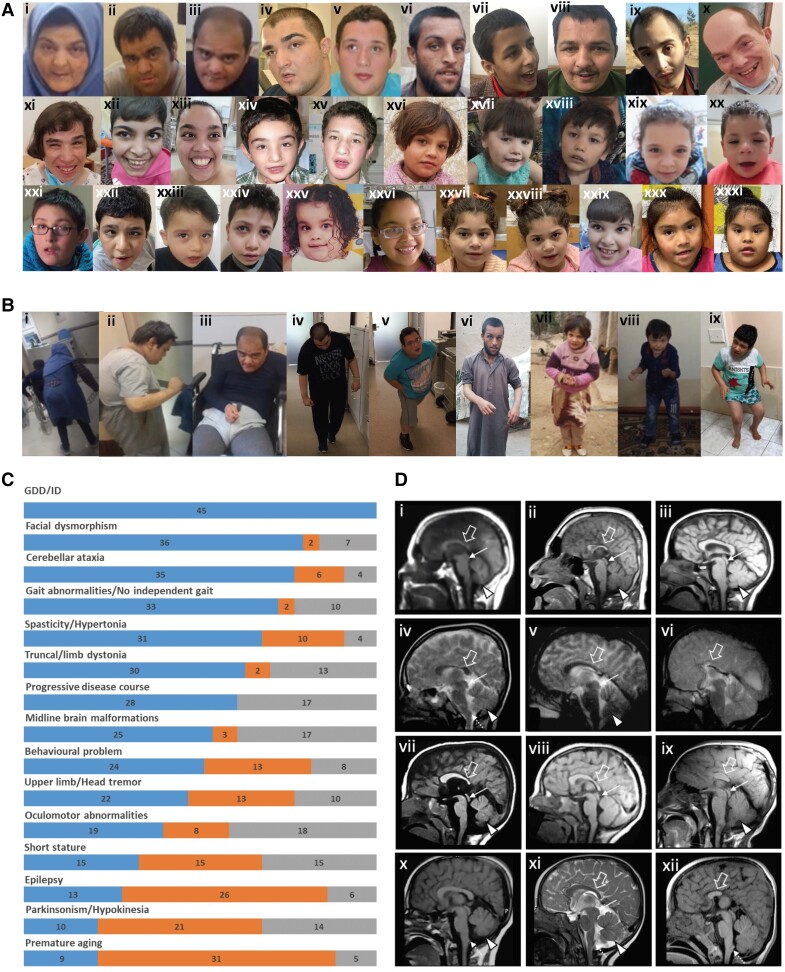
**Clinical features of the affected individuals with bi-allelic *ACBD6* variants**. [**A**(**i**–**xiii**)] Representative photographs demonstrating facial features of the affected individuals in adulthood: F1:S1 (**i**); F1:S2 (**ii**); F1:S3 (**iii**); F3:S2 (**iv**); F3:S1 (**v**); F6:S1 (**vi**); F7:S2 (**vii**); F7:S1 (**viii**); F11:S1 (**ix**); F17:S1 (**x**); F17:S2 (**xi**); F20:S1 (**xii**); and F16:S1 (**xiii**). [**A**(**xiv**–**xxxi**)] Representative photographs demonstrating facial features of the affected individuals in childhood: F3:S2 (**xiv**) at 5 years old; F3:S1 (**xv**) at 12 years old; F6:S2 (**xvi**); F8:S1 (**xvii**); F8:S2 (**xviii**); F10:S1 (**xix**); F10:S2 (**xx**); and F11:S1 (**xxi**) at younger age; F13:S1 (**xxii**); F14:S1 (**xxiii**); F15:S1 (**xxiv**); F16:S1 (**xxv**) at 2 years old; F16:S1(**xxvi**) at 4 years old; F19:S1 (**xxvii**); F19:S2 (**xxviii**); F20:S2 (**xxix**); F12:S1 (**xxx**); and F12:S2 (**xxxi**). The most frequently seen facial dysmorphologies in adults are high nasal ridge, full nasal tip, small mouth, thin upper lip and broad chin. The most frequently seen facial dysmorphologies in children are bifrontal/bitemporal narrowing, arched eyebrows, hypertelorism, up-slanting palpebral fissures, depressed nasal bridge, full nasal tip, thin upper lip, full lower lip and broad chin. [**B**(**i**–**ix**)] Representative photographs demonstrating postural abnormalities seen in the affected individuals. A stooped body posture and lateral flexion of the trunk can be seen in the individuals F1:S1 (**i**), F1:S2 (**ii**), F1:S3 (**iii**), F3:S1 (**iv**), F3:S2 (**v**), F6:S1 (**vi**), F6:S2 (**vii**), F8:S2 (**viii**) and F13:S1 (**ix**). (**C**) Bar graph summarizing proportions of various clinical findings in the *ACBD6* cohort. Blue = affected; orange = unaffected; grey = not ascertained/not applicable. GDD = global developmental delay; ID = intellectual disability. [**D**(**i**–**xii**)] Representative neuroimaging features of the affected individuals. Brain MRI, midline sagittal images of the affected individuals F1:S1 (**i**), F1:S2 (**ii**), F5:S1(**iii**), F56:S2 (**iv**), F7:S1 (**v**), F7:S2 (**vi**), F10:S1 (**vii**), F10:S2 (**viii**), F11:S1 (**ix**), F13:S1 (**x**), F14:S1 (**xi**) and F19:S1 (**xii**). Most of the affected individuals have corpus callosum agenesis or hypoplasia with prevalent involvement of the posterior sections (empty arrows), variably associated with short midbrain (thin arrows) and small inferior cerebellar vermis (arrowheads). In addition, mild hypertrophy of the clava was noted in some subjects (dotted arrows). Note that the anterior commissure was markedly hypoplastic or absent in all affected individuals.

The cohort includes 23 male and 22 female affected individuals whose current ages widely range between 1 and 50 years. While almost half of the individuals (21/45) were <10 years old (the first age group), 11/45 (25%) individuals were between the ages of 10 and 19 years (the second age group) and 13/45 (29%) affected individuals were ≥20 years old (the third age group) at the most recent review. Two siblings from Family 28 died at the ages of 31 and 30 due to stage 5 chronic renal disease and aspiration, respectively. Almost all affected individuals are from consanguineous unions with diverse ethnic backgrounds populating South and Central Asia, the greater Middle East, Europe, and North and South America. Table 1 and Fig. 2A–D provide a summary of the core phenotypic features of 45 affected individuals from 28 unrelated families with bi-allelic *ACBD6* variants. A detailed phenotypic description is provided in Supplementary Table 1 and the Supplementary material, ‘Case reports’ section. Video recordings are available from 16 families (Supplementary Videos 1–16, available at https://doi.org/10.6084/m9.figshare.25436116.v1).

Prenatal history was mostly unremarkable in the cohort and most of the affected individuals reached normal gestational age. Head circumference at birth was ≤3rd percentile in 7/21 (33%) affected individuals. Head circumference on the latest available review was ≤2nd percentile in 12/31 (39%) individuals and height was mostly below the age-adjusted average in the cohort (18/32, 56%). Current weight was >50th percentile in 20/34 (59%) affected individuals. All patients manifested a moderate-to-severe global developmental delay (GDD) involving all domains but predominantly affecting cognitive function and the acquisition of independent walking and expressive language. While 10 patients had failed to acquire independent ambulation by a mean age of 9.4 ± 5.0 years (age range 4–20), the mean age of independent walking for the rest of the cohort was 3.7 ± 1.7 years (age range 1.5–8).

Upon the latest available follow-up, moderate-to-severe GDD/ID (45/45, 100%) with significant expressive language impairment (40/41, 98% from non-verbal to a few words), movement disorders (33/34, 97%), facial dysmorphism (38/40, 95%) and mild cerebellar ataxia (35/41, 85%) associated with limb spasticity/hypertonia (31/41, 76%) and gait abnormalities (33/35, 94%) were among the cardinal clinical features observed in the *ACBD6* cohort (Fig. 2C).

The most conspicuous and common movement disorder present in the three age groups was dystonia (30/32, 94%). This was frequently truncal dystonia leading to abnormal thoracic and/or thoracolumbar spinal flexion (camptocormia) (30/31, 97%) and mild-to-moderate lateral flexion of the trunk (Pisa syndrome, 22/32, 69%). Although the stooping of the body and its lateral flexion were equally common in the second (9/9 and 8/9, respectively) and the third (10/10 and 10/10, respectively) age groups, and were frequent in the first age group (10/12 and 4/12, respectively), the severity of truncal dystonia suggested an age-dependent progression (Fig. 2B). Additionally, some affected individuals developed mild upper limb action-induced dystonia (6/15, 40%), lower limb dystonia (12/22, 55%) and cervical dystonia (8/26, 31%).

Another common hyperkinetic movement disorder in the cohort was a tremor. The upper limb jerky tremor at rest and/or intention tremor was present in 22/35 (63%) affected individuals and 16/27 (59%) individuals had a mild head tremor. Dystonic head tremor, jerky tremor involving all limbs and negative myoclonus were also seen in a small number of patients. With advancing age, parkinsonism/hypokinesia developed in 10/31 (32%) individuals, six of whom were over the age of 20 years, and four were between 10 and 20 years of age. A trial of levodopa was done in only two affected individuals with a minimal response. No other antiparkinsonian or anti-dystonic medication has been tried in the cohort. Additionally, subtle perioral muscle twitching and stereotypic mouth dyskinesia were observed in the available video recordings of younger affected individuals. Remarkably, simple motor and vocal tics and tic-like vocalizations were detected in the video recordings of seven young and adult cases (Supplementary Videos 1, 2, 6, 9, 12 and 14–16, available at https://doi.org/10.6084/m9.figshare.25436116.v1). Regarding eye movements, limited upgaze (12/26, 46%), impaired saccades (9/19, 47%) and strabismus (8/20, 40%) were frequent oculomotor abnormalities.

Lower limb spasticity, ascertained in 27/35 (77%) affected individuals, in combination with cerebellar ataxia led to gait abnormalities described as a spastic-ataxic gait in 14 patients, and clumsy/slow/broad-based or unstable gait in 17 individuals. Upper limb ataxia and spasticity were confirmed in 13 and nine affected individuals, respectively. Tendon release surgery was done in three cohort members due to lower limb spasticity. Lower limb hypotonia was detected in single isolated cases.

A deterioration in motor and cognitive abilities was reported in 28/28 (100%) affected individuals, suggesting a progressive disease course and underlying neurodegeneration. The oldest member of the cohort, currently aged 50 years, has lost his ability to walk independently and currently uses a wheelchair.

Complex partial, myoclonic, atonic and generalized tonic-clonic seizures were reported in 13/39 (33%) patients, with the seizure debut from neonatal to 35 years of age. Seizures were often controlled with a combination of antiepileptic medications.

Younger affected individuals were reported to have hand stereotypies, and hyperactivity was present in 9/21 (43%) patients. Signs of premature ageing were seen in 9/38 (24%) individuals from five families. Forty-six per cent of patients had autistic features (13/28), temper tantrums 16/36 (44%) and aggressive behaviour (13/37, 35%) with a tendency to self-injury (6/34, 18%). Sleep disturbances (15/33, 45%) were common, and urinary incontinence was present in 15/24 (63%) individuals aged between 3 and 20 years.

Facial photographs were available from 32 affected individuals from 19 families (Fig. 2A). The analysis of 19 children revealed the most frequent dysmorphic features, including full nasal tip (16/19), broad chin (14/19), bifrontal/bitemporal narrowing (12/19), hypertelorism (11/19), up-slanting palpebral fissures (9/19) and depressed nasal bridge (9/19). Assessment of 13 adult photos showed frequent signs such as a broad chin (11/13), full nasal tip (8/13), small mouth (7/13), high nasal ridge (5/13), thin upper lip (5/13) and full lower lip (5/13) (more details in Supplementary Tables 3 and 4).

Brain MRI scans were available for neuroradiological review in 29/45 subjects. The corpus callosum was abnormal in 20/29 subjects (70%): partial or complete callosal agenesis was observed in seven individuals, while callosal hypoplasia with prevalent involvement of the posterior sections was noted in the remaining 13 individuals. In 19/29 patients (66%), there was marked hypoplasia/agenesis of the anterior commissure. Short midbrain and small inferior cerebellar vermis were each detected in 11/29 affected individuals (38%). Mild to moderate reduction of periventricular white matter with consequent ventriculomegaly was observed in 10/29 patients (34%). In 7/29 (24%) individuals, incomplete hippocampal inversion was found. Finally, mild hypertrophy of the clava was noted in 5/21 (24%) individuals (Fig. 2D). Only 4/29 patients (14%) had normal neuroimaging findings.

### *Acbd6* expression studies in mouse brain

*Acbd6* is expressed in nearly all regions of the adult mouse brain profiled by single-cell RNA sequencing (Supplementary Fig. 3).

### Characterization of *ACBD6* LOF using CRISPR/Cas9-mediated zebrafish mutant and F_0_ knockout models

**Figure 3:**
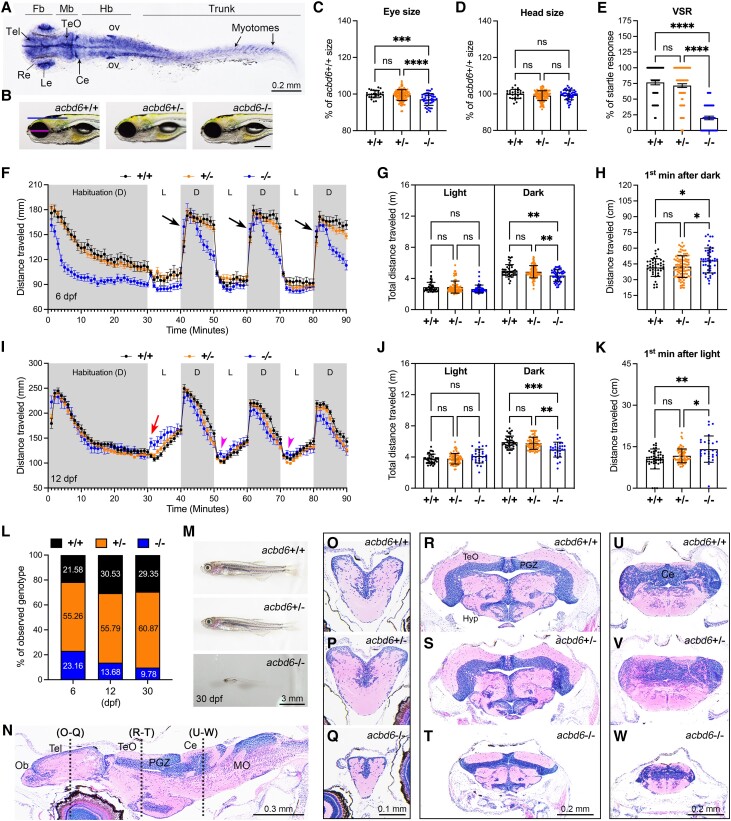
**CRISPR/Cas9 mutation of zebrafish *acbd6* causes smaller eyes, impaired vision, abnormal locomotion, developmental delay and increased mortality**. (**A**) Whole-mount *in situ* hybridization for detecting *acbd6* mRNA expression pattern in zebrafish embryo at 24 hours post-fertilization (hpf). fb = forebrain; mb = midbrain; MHB = midbrain and hindbrain boundary; hb = hindbrain; ov = otic vesicle. Dorsal view to the *top*, anterior to the *left*. (**B**) Representative images of wild-type (*acbd6*+/+), heterozygous (*acbd6*+/−) and homozygous (*acbd6*−/−) mutant larva at 6 days post-fertilization (dpf). Head size and eye size are indicated by blue and red lines, respectively. Anterior to the *left* and dorsal to the *top*. Scale bar = 200 μm. (**C** and **D**) Quantification of eye and head size as indicated in **B**. +/+ (*n* = 26 larvae), +/− (*n* = 114 larvae) and −/− (*n* = 47 larvae). Each symbol represents one larva. Values are calculated as a percentage of the mean value of +/+ larvae. Error bars = mean ± standard deviation (SD). (**E**) The result of visual startle response analysis performed on +/+ (*n* = 43 larvae), +/− (*n* = 99 larvae) and −/− (*n* = 48 larvae) zebrafish larvae at 6 dpf. Each symbol represents one larva. The number of responses for five stimuli of each larva is calculated as a percentage of responses. Error bars = mean ± standard error of the mean (SEM). (**F**) Locomotor activities of zebrafish larvae in light and dark periods at 6 dpf. +/+ (*n* = 42 larvae), +/− (*n* = 99 larvae) and −/− (*n* = 48 larvae) zebrafish larvae were habituated in the dark for 30 min, followed by three cycles of 10-min time bins of light and dark periods. Black arrows indicate the increased movement of homozygous mutants at the first minute in the dark. Error bars = mean ± SEM. D = dark period; L = light period. (**G**) Average cumulative distance travelled by each larva from three cycles of either light or dark periods in **F**. Error bars = mean ± SD. (**H**) Average cumulative distance travelled by each larva during the first minute of the dark period across three cycles as indicated by black arrows in **F**. Error bars = mean ± SD. (**I**) Locomotor activities of zebrafish larvae in light and dark conditions at 12 dpf. +/+ (*n* = 39 larvae), +/− (*n* = 71 larvae) and −/− (*n* = 29 larvae). Error bars = mean ± SEM. Red arrow indicates increased movement of homozygous mutants at the first minute after light on. Red arrowhead indicates increased movement of homozygous mutants at the second minute after light on. (**J**) Average cumulative distance travelled by each larva during three cycles of either light or dark periods in **I**. Error bars = mean ± SD. (**K**) Average cumulative distance travelled by each larva during the first cycle of the first minute of the light period as indicated by red arrow in **I**. Error bars = mean ± SD. (**L**) Genotyping results of zebrafish at 6 dpf (*n* = 191 larvae), 12 dpf (*n* = 196 larvae) and 30 dpf (*n* = 118 juveniles) stages from *acbd6*+/− intercross. (**M**) Representative images of morphological phenotype from *acbd6*+/+, *acbd6*+/− and *acbd6*−/− at 30 dpf. Anterior to the *left* and dorsal to the *top*. (**N**) Sagittal section of *acbd6*+/+ brain at 30 dpf. Anterior to the *left* and dorsal to the *top*. MO = medulla oblongata; Ob = olfactory bulb; PGZ = periventricular grey zone of optic tectum. (**O**–**W**) Representative images of transverse sections of telencephalon (**O**–**Q**), optic tectum (**R**–**T**) and cerebellum (**U**–**W**) from *acbd6*+/+, *acbd6*+/− and *acbd6*−/− juvenile as indicated in **N**. In **C** and **D**, one-way ANOVA with Tukey’s multiple comparisons test; in **E**, **G**, **H**, **J** and **K**, one-way ANOVA with Dunnett’s T3 multiple comparisons test; ns, not significant; *P < 0.05; **P < 0.01; ***P < 0.001; ****P < 0.0001.

Zebrafish Acbd6 protein is highly conserved across species and displays similar tissue-specific expressions to humans (Supplementary Fig. 4). To examine the spatiotemporal expression pattern, we performed whole-mount *in situ* hybridization (WISH) analysis, and the results revealed that the *acbd6* mRNA was broadly expressed at 24 h post-fertilization (hpf) and had elevated expression levels in the CNS, developing eyes, otic vesicle and trunk muscles (Fig. 3A). Using CRISPR/Cas9 technology, we generated a genetic mutant of *acbd6*, and through real-time quantitative PCR analysis, verified a significant notable decrease in *acbd6* mRNA expression in homozygous mutants (Supplementary Fig. 5A). During early embryonic developmental stages, we did not observe any visible morphological abnormalities in homozygous mutants. However, at the 6 days post-fertilization (dpf) stage, homozygous mutants (−/−) demonstrated a minor reduction in eye size (∼3%, indicated by the red line in Fig. 3B) compared with wild-type (+/+) or heterozygous (+/−) animals (Fig. 3C). There was no significant change in head size (indicated by the blue line in Fig. 3B) (Fig. 3D). The visual startle response analysis^24^ indicated that reduced eye size impacts the visual function in *acbd6*^−/−^ mutants (Fig. 3E). Furthermore, we performed locomotion behaviour tests on mutants at 6 and 12 dpf in 10-min intervals of light-dark cycles (Fig. 3F–K and Supplementary Figs 5–7, with detailed descriptions in the Supplementary material, ‘Result 3’ section). In general, *acbd6*^−/−^ mutants exhibited a gradual decline in locomotor activity in dark periods (Fig. 3F, G, I and J and Supplementary Fig. 7A, B, D and E) and an exaggerated response as soon as lights are turned off at 6 dpf (Fig. 3F and H and Supplementary Figs 5C and D and 7A and C), suggesting a hypertonia-like or spasticity behaviour.^25^ As the larvae developed further at 12 dpf, the *acbd6^−/−^* mutants showed an increase in distance moved after light on and multiple locomotor bursts (Fig. 3I and K and Supplementary Figs 6C and 7D and F), indicating light-induced seizure-like behaviour.^26^ The mutants also demonstrated increased mortality and severe developmental delay, with an overall reduction in brain size and disrupted muscular phenotype (Fig. 3L and M). Histological analysis showed a reduction in brain size (Fig. 3N–W), particularly in regions such as the telencephalon, optic tectum, cerebellum and retina (Fig. 3O–Q, R–T and U–W, respectively, and Supplementary Fig. 8A–C). Furthermore, skeletal muscle fibres in *acbd6^−/−^* mutants displayed a disrupted phenotype, characterized by shortened and scattered fibres and gaps (Supplementary Fig. 8D–F). Interestingly, adult *acbd6*^−/−^ mutant survivors (Supplementary Fig. 9 and Supplementary Videos 17 and 18; videos available at https://doi.org/10.6084/m9.figshare.25436116.v1) exhibited behaviour resembling that of individuals with an autism spectrum disorder.^27^

**Figure 4:**
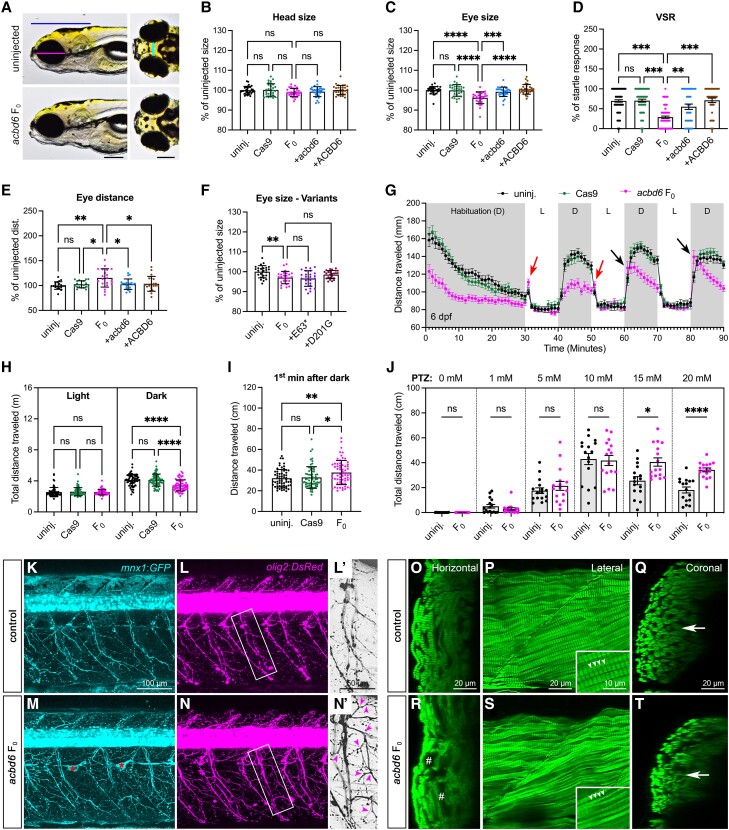
**Zebrafish *acbd6* F_0_ knockouts exhibit increased susceptibility to chemical-induced seizures, excessive motor neuron branching and skeletal muscle degeneration**. (**A**) Representative images of uninjected control and *acbd6* F_0_ larvae at 6 days post-fertilization (dpf). *Left*: Ventral view, anterior to the left. *Right*: Dorsal view, anterior to the left. Blue line indicates head size; magenta line indicates eye size; and cyan line indicates eye distance. Scale bar = 0.2 mm. (**B** and **C**) Quantification of the head and eye size (*n* = 30 larvae for each group) of uninjected control (uninj.), *acbd6* F_0_ knockout (F_0_), F_0_ + zebrafish wild-type *acbd6* mRNA (+acbd6) and F_0_ + human wild-type *ACBD6* mRNA (+ACBD6) as indicated in **B**. (**D**) The visual startle response (VSR) analysis after mRNA rescue at 6 dpf. *n* = 36 larvae for each group. Each symbol represents one larva. The number of responses to five stimuli of each larva was calculated as a percentage of responses. Error bars = mean ± standard error of the mean (SEM). (**E**) Quantification of the eyes distance (*n* = 20 larvae for each group) as indicated in **B**. (**F**) Quantification of the eye size of F_0_ knockout rescued with mRNA of human p.Glu63Ter (+E63*) or p.Asp201Gly (+D201G) variant. *n* = 25 larvae for each group. (**G**) Locomotor activities of zebrafish larvae in light and dark conditions at 6 dpf. *n* = 64 larvae for each group. The larvae were habituated in the dark for 30 min, followed by three cycles of 10-min periods of light and dark. Error bars represent the mean ± SEM. D = dark period; L = light period. Red arrows indicate increased movement of F_0_ 1 min after light on, and black arrows indicate increased movement 1 min after light off. (**H**) Average cumulative distance travelled by each larva during three cycles of either light or dark periods in **G**. Error bars = mean ± standard deviation (SD). (**I**) The average cumulative distance traveled by each larva during the first minute of the dark period was measured over three cycles, as shown by the black arrow in **G**. Error bars represent the mean ± SD. (**J**) The average cumulative distance traveled by the larvae was measured for each group after being treated with different doses of pentylenetetrazole (PTZ) at 5 dpf. *n* = 16 larvae for each group. (**K**–**N**) Confocal images of *Tg(mnx1:GFP; olig2:DsRed)* larva at 12 dpf are shown, with transgenic larvae injected with *slc45a2* sgRNA used as a control and those injected with *acbd6* + *slc45a2* sgRNAs shown as *acbd6* F_0_. (**L'** and **N'**) Enlarged images from white boxes are shown in **L'** and **N'**, with red asterisks indicating autofluorescence from remaining pigment cells. GFP and DsRed are displayed in cyan and magenta, respectively, with magenta arrowheads indicating excess axonal arborizations. The images are presented in a lateral view, with anterior to the *left* and dorsal to the *top*. Additional motor neuron phenotypes at 6 and 12 dpf can be found in Supplementary Fig. 1^3^. (**O**–**T**) Confocal images of stained skeletal muscle fibers with phalloidin are presented, including images from *slc45a2* sgRNA-injected control (**O**–**Q**) and *acbd6* + *slc45a2* sgRNA-injected (**R** and **S**) larvae at 12 dpf. Orthogonal views generated from **P** and **S** using the Orthogonal views tool in ImageJ are also displayed. ^#^Degenerated muscles. White arrowheads and a white arrow indicate Z-discs and the thickness of the myotube, respectively. Supplementary Fig. 1^4^ provides additional muscle phenotypes at 6 and 12 dpf. In **B**–**D** and **F**, one-way ANOVA with Tukey’s multiple comparisons test; **E** and **H**–**J**, one-way ANOVA with Dunnett’s T3 multiple comparisons test; ns, not significant; *P < 0.05; **P < 0.01; ***P < 0.001; ****P < 0.0001.

To verify the specificity of the *acbd6* mutant phenotype, we used the F_0_ knockout (also known as F_0_ crispant, or simply F_0_) method to induce bi-allelic mutations and observed similar morphological and molecular phenotypes in homozygous mutants and F_0_ (Fig. 4A–D). We discovered that F_0_ also exhibited several phenotypes previously reported in affected individuals, such as hypertelorism (Fig. 4E) and facial dysmorphism (broader chin and wider lower jaw; Supplementary Fig. 10A–H). These phenotypes were restored upon co-injection of wild-type human mRNA, confirming the specificity of the phenotype. We also introduced the LOF p.(Glu63Ter) and missense variants p.(Asp201Gly) into *acbd6* cDNA and observed impaired protein function and failure to rescue the eye size phenotype (Fig. 4F) in F_0_ knockouts. We further investigated *acbd6* F_0_ behaviour in light-dark cycles at 6 and 12 dpf and observed that *acbd6* F_0_ exhibited lower locomotor activity during dark periods (Fig. 4G and H) and an exaggerated response after lights off, like homozygous mutants (Fig. 4G and I and Supplementary Fig. 11A and B). Additionally, *acbd6* F_0_ exhibited light-induced seizure-like behaviour (red arrows in Fig. 4G and Supplementary Fig. 11C and D). By 12 dpf, *acbd6* F_0_ demonstrated reduced locomotor activity in both light and dark periods (Supplementary Fig. 12A and B). These results suggest that *acbd6* F_0_ larvae exhibit more severe abnormal locomotor behaviours than homozygous mutants. We hypothesized that the loss of *acbd6* might increase susceptibility to chemical-induced seizures, as observed in *acbd6*^*−/−*^ mutants showing seizure-like behaviour.^28,29^ To test this hypothesis, we exposed *acbd6* F_0_ and control larvae to different doses of the anticonvulsant drug, pentylenetetrazole (PTZ), and discovered that *acbd6* F_0_ larvae exhibited hyperexcited behaviour at higher doses (Fig. 4J). This suggests that downregulation of *acbd6* may contribute to the onset of epilepsy-like seizures. We examined the impact of *acbd6* F_0_ on neuronal and skeletal muscle development and discovered excessive axonal arborizations (Fig. 4K–N and Supplementary Fig. 1^3^) and progressive degeneration of muscle fibres in *acbd6* F_0_ larvae (Fig. 4O–T and Supplementary Fig. 14). We also observed an increase in myelin basic protein a (*mbpa*) expression in both *acbd6* F_0_ and homozygous mutant (Supplementary Fig. 15A and B), which may explain the abnormal axonal development phenotype. A detailed description of our results can be found in the Supplementary material, ‘Result 2’ section. In summary, our zebrafish model replicated many of the clinical features seen in individuals with bi-allelic variants in *ACBD6*, highlighting how these variants may contribute to the progressive disease course. Our extensive analysis of both mutant and F_0_ in *acbd6* provides insight into the underlying mechanisms of the disease observed in affected individuals.

### *Xenopus tropicalis* ACBD6 F_0_ knockout models

**Figure 5:**
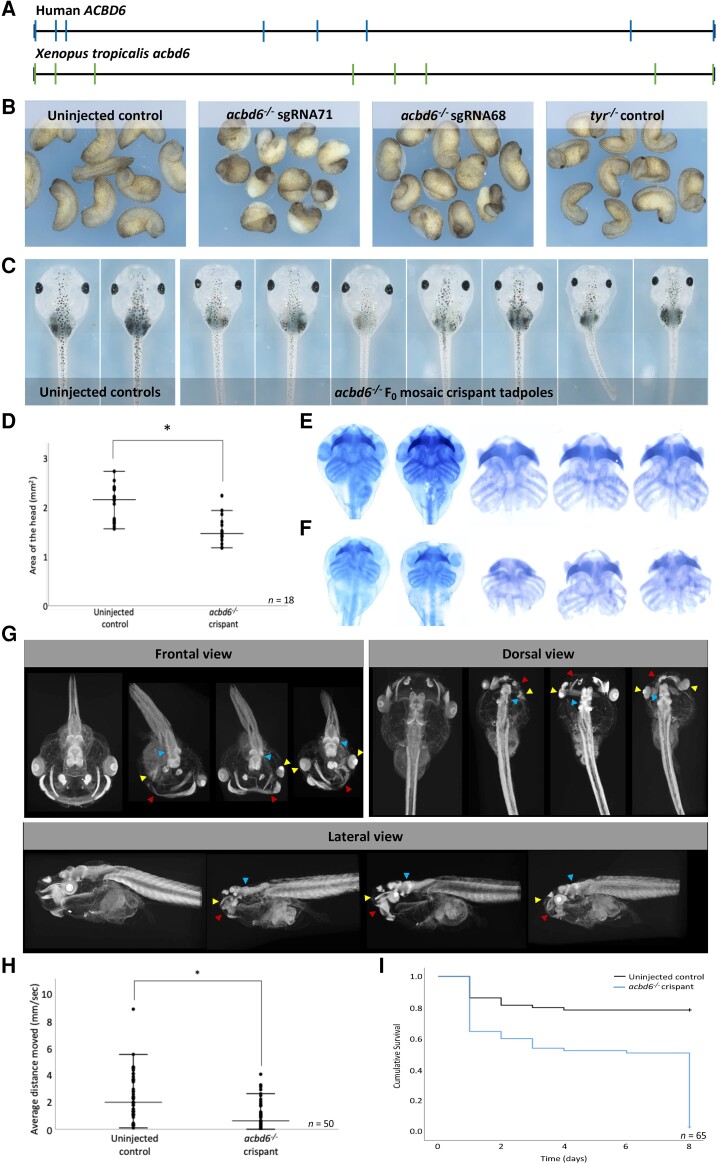
***Xenopus tropicalis* (*acbd6*) crispants have gastrulation, movement, craniofacial, brain and eye defects together with microcephaly**. (**A**) The gene structure of human (*ACBD6*) and *X. tropicalis* (*acbd6*) reveals eight exons. (**B**) Gastrulation defects, including failure of blastopore closure and anterior posterior defects, were observed in F_0_*X. tropicalis* embryos injected with two different CRISPR/Cas9 constructs (sgRNA-68 and sgRNA-71) disrupting exon 1 of *acbd6*. (**C**) Those animals surviving to free-feeding stages presented with microcephaly, craniofacial dysmorphism and eye abnormalities. (**D**) The differences in head size between the uninjected control (2.07 ± 0.36 mm) and *acbd6* crispant tadpoles (1.52 ± 0.27 mm, sgRNA-68) were found to be significant, *t*(34) = 5.183, *P* < 0.001. (**E** and **F**) Alcian blue staining marking the cartilaginous structures in the head and neck show equivalent structures between control (**E**) and *acbd6* crispant tadpoles (**F**), revealing no gross morphological abnormalities. (**G**) Detailed structural analysis in higher resolution microCT imaging (1% phosphotungstic acid contrast stain) revealed significant structural abnormalities in the facial musculature (red arrows; **G**), abnormalities of the eye (microphthalmia, anophthalmia; yellow arrows, **G**) and structural abnormalities in the brain most pronounced in the midbrain regions (blue arrow, **G**). (**H**) Locomotion analysis at NF44/45 revealed that crispants moved significantly less than control tadpoles. (**I**) The Kaplan–Meier survival analysis of 65 control and crispant tadpoles shows two periods of crispant-specific decline, the first at gastrula stages (Day 0–1) and the second with post-feeding [Day 8, Nieuwkoop and Faber (NF) stage 47].

*Xenopus tropicalis* and humans have the same *acbd6* gene structures (Fig. 5A) and share 66% amino acid identity (Supplementary Fig. 16A). Crispant F_0_ tadpoles were produced by injection of two non-overlapping sgRNAs targeting exon 1 of *acbd6* (Supplementary Fig. 16B). The effects were specific, since both sgRNAs produced the same phenotype and when embryos with a single nucleotide polymorphism in the PAM for one sgRNA were used, the embryos developed normally (Supplementary Fig. 17A, third upper panel from the left). Inference of CRISPR edits (ICE) analysis^30^ of target amplicon sequences (Supplementary Fig. 16C) showed that at the gastrula stage, 74% of alleles in the embryos had indels, and that 63.5% had a frameshift from a predominant 8 bp deletion (sgRNA 68). For sgRNA71, the average was a 53.6% knockout from a mix of indels. The range of phenotypes at later stages was due to distinct levels of frameshift mutations among the groups (Supplementary Fig. 16D).

The first notable phenotype was gastrulation failure due to reduced cell movements (Fig. 5B and Supplementary Fig. 17A). This limits the analysis of phenotypes at later stages since the surviving embryos have been selected to have significantly greater mosaicism than normally produced in this type of experiment. At swimming tadpole stages more than half of the crispants had obvious craniofacial abnormalities (*n* = 36; Fig. 5C and Supplementary Fig. 17D and E) and head measurement showed a decrease in the average area from 2.07 ± 0.36 mm^2^ in controls to 1.52 ± 0.27 mm^2^ in crispants [*t*(34) = 5.183, *P* < 0.001; Fig. 5D]. This was not a result of a defect in the structure of the head cartilage, although when *in situ* it did appear constrained by the overall head structure [*cf*. Fig. 5E (control) and F (crispant)]. The cartilage components were nonetheless present and morphologically normal upon dissection; however, the overall cartilage size was smaller than controls consistent with the observed microcephaly. To detect subtler changes in head structure, we compared control and crispant tadpoles by microCT (Fig. 5G). Three things were apparent in the three embryos selected at random from the crispant group: first, the eyes (yellow arrows) were abnormal and displaced (e.g. the dorsal and frontal views in the right panel) and in one case there was anophthalmia; the latter was rare because it was not detected in the bright field images. Second, a muscle in the face was poorly developed or absent (red arrows). From comparison with *X. laevis* staining,^31^ we tentatively identified this as the geniohyoidius, although the muscle seems more distant from the midline in *X. tropicalis* than in *X. laevis*. The brain also has obvious structural abnormalities which are most pronounced in the midbrain (blue arrows). Comparing the movement of control and *acbd6* crispant tadpoles showed that the crispants move less over a 10-min period (average 2.37 mm/s for controls and 1.01 mm/s for crispants, *n* = 50). The difference was statistically significant [*t*(78) = 4.9, *P* ≤ 0.001] (Fig. 5H and Supplementary Videos 19–22; videos available at https://doi.org/10.6084/m9.figshare.25436116.v1). After the deaths at gastrulation, crispants and control embryos survived similarly until the feeding stage (Fig. 5I).

### Loss of ACBD6 does not impact peroxisome function

**Figure 6:**
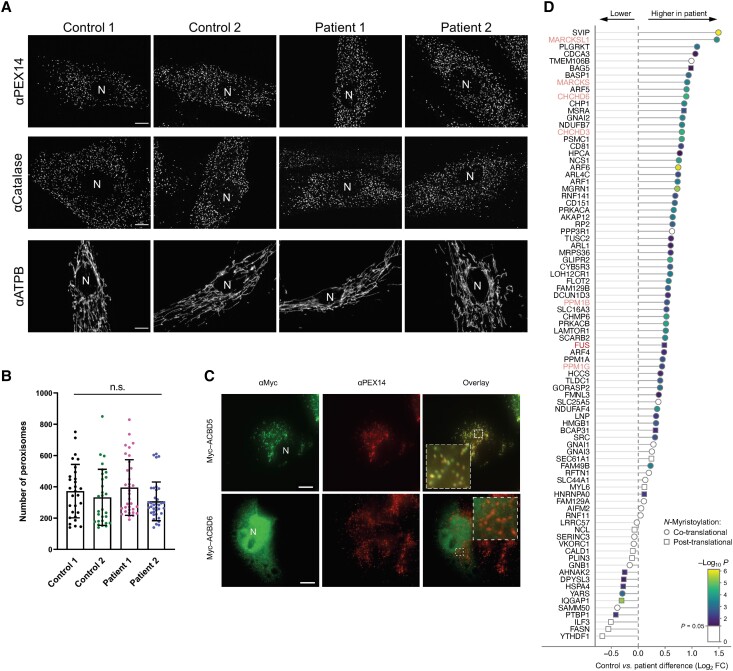
**Morphological characteristics of peroxisomes in *ACBD6*-deficient patient cells are not altered and chemical proteomic profiling of *N*-myristoylation in human fibroblasts**. (**A**) Patient fibroblasts and controls were processed for immunofluorescence microscopy using antibodies against the peroxisomal membrane marker PEX14, the matrix marker catalase or mitochondrial ATP synthetase B (ATPB). Peroxisomal localization of PEX14 and catalase indicate that peroxisomal membrane and PTS1-dependent matrix import are normal. Note that the morphology of mitochondria, which are elongated in fibroblasts, was also not altered when compared to controls. (**B**) Quantification of peroxisome number based on immunofluorescence images (see **A** for representative images) (*n* = 29–36 cells). Data are from three independent experiments. ns, not significant; Kruskal–Wallis ANOVA test with Dunn’s multiple comparisons. (**C**) COS-7 cells were transfected with plasmids encoding Myc-ACBD5 or Myc-ACBD6 and processed for immunofluorescence microscopy using antibodies against Myc and PEX14. Note that Myc-ACBD5 localizes to peroxisomes, whereas Myc-ACBD6 localizes to the nucleus and the cytoplasm in COS-7 cells. Scale bars = 10 µm. (**D**) Ranked plot of myristic acid alkyne (YnMyr)-labelled, known co- and post-translationally *N*-myristoylated proteins, as identified in Supplementary Fig. 18E. Position on the *left* equals lower abundance in ACBD6 deficient fibroblasts, position on the *right* equals higher abundance in ACBD6 deficient fibroblasts.

Several members of the ACBD family have been linked to peroxisome function.^2^ Furthermore, peroxisomal dysfunction is linked to developmental defects and neurological abnormalities.^32^ To investigate if peroxisomal parameters were altered in *ACBD6* deficiency, patient (from F1:S1 and F2:S1) and control (F1:II-2 unaffected sibling; wild-type C109) fibroblasts were processed for immunofluorescence using antibodies against the peroxisomal membrane marker PEX14 and catalase, a peroxisomal matrix protein (Fig. 6A and B). No alterations in peroxisome protein import, morphology, distribution, or number were observed when compared to control fibroblasts (Fig. 6A and B). In addition, expression of a Myc-ACBD6 construct in COS-7 cells confirmed a cytoplasmic and nuclear localization of Myc-ACBD6^33^ but did not provide evidence for a peroxisomal localization under standard culture conditions (Fig. 6C). In addition, fatty acid analysis after a D3-C22:0 loading test in cultured fibroblasts did not reveal any abnormalities of peroxisomal very long-chain fatty acid β-oxidation (Supplementary Table 5) as observed in *ACBD5* deficiency.^5,32^ Detailed results are described in the Supplementary material, ‘Results 3’ section.

### Investigation of the effect of *ACBD6* deficiency on *N*-myristoylation in patient-derived fibroblasts

We employed a chemical proteomic approach to identify and quantify *N*-myristoylated proteins, by combining metabolic labelling of living cells with YnMyr,^14^ an alkyne-containing myristic acid analogue, and Click chemistry-enabled enrichment of YnMyr-labelled proteins, coupled to mass spectrometry proteomics analysis.^22^ Chemical proteomics revealed 68 known co-translationally and 18 post-translationally *N*-myristoylated proteins expressed in the fibroblasts derived from F1:S3 and his healthy sibling (Supplementary Fig. 18A and B, respectively). As expected, the incorporation of YnMyr was markedly reduced by treatment with 100 nM IMP-1088, a selective and highly potent NMT inhibitor,^23,34^ in both the fibroblasts of the healthy control and the patient, confirming the specificity of labelling (Supplementary Fig. 18C and D, respectively). Proteins co- and post-translationally *N*-myristoylated with YnMyr were detected in significantly higher abundance in the patient-derived fibroblasts compared to the healthy sibling (Supplementary Fig. 18E), suggesting ACBD6 deficiency provokes increased incorporation of YnMyr, potentially through increased *N*-myristoylation or upregulation of lipid salvage pathways leading to increased YnMyr import (Fig. 6D). We hypothesize this may be caused by the differential interplay of NMT1 and NMT2 with ACBD6 for various substrates. Although fibroblasts from only one affected individual and a healthy sibling were compared, the apparent differential *N*-myristoylation warrants more detailed investigations of the role of ACBD6 in *N*-myristoylation in cell types involved in the described human clinical phenotypes.

### Proteomics analyses of developing wild-type and *acbd6* crispant zebrafish

**Figure 7:**
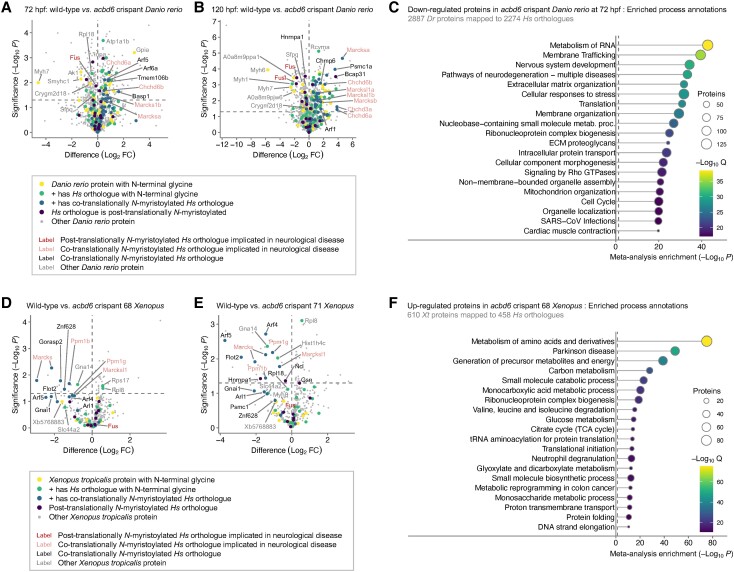
**Chemical and whole proteome analysis of *acbd6* wild-type and crispant zebrafish and *Xenopus tropicalis* model systems**. (**A**) Volcano plot comparing myristic acid alkyne (YnMyr) labelling of proteins in wild-type and *acbd6* crispant zebrafish at 72 h post-fertilization (hpf). (**B**) Comparing YnMyr labelling of proteins in wild-type and *acbd6* crispant zebrafish at 120 hpf. Further description as in **A**. (**C**) Top 20 biological processes most significantly enriched in proteins downregulated in *acbd6* crispant zebrafish at 72 hpf. (**D**) Volcano plot comparing YnMyr labelling of proteins in wild-type and *acbd6* crispant 68 *X. tropicalis*. (**E**) Comparing YnMyr labelling of proteins in wild-type and *acbd6* crispant 71 *X. tropicalis.* Further description as in **D**. (**F**) Top 20 biological processes most significantly enriched in proteins upregulated in *acbd6* crispant 68 *X. tropicalis*. Most significantly enriched processes are at the *top*. In **A** and **D**, position on the *left* equals reduced in crispant; position on the *right* equals increased in crispant. Horizontal dotted line indicates significance threshold (*P* = 0.05). FC = fold-change. In **C** and **F**, the most significantly enriched process is at the *top*. Colour indicates Q-value as secondary significance indicator. Size of circle indicates a number of proteins enriched in the depicted process. Dr = *Danio rerio*; Hs = *Homo sapiens*; Xt = *X. tropicalis*.

To shed light on the potential role of *acbd6* on *N*-myristoylation during zebrafish development, we employed metabolic labelling with YnMyr and chemical proteomics at different stages of development. At 72 and 120 hpf, both wild-type and *acbd6* crispant zebrafish express >32 significantly enriched proteins, for each of which the human orthologue is a validated co-translationally *N*-myristoylated substrate (Supplementary Fig. 19A–D). In addition, significantly enriched proteins also included >48 proteins with N-terminal glycine, thereby potentially *N*-myristoylated, but where unequivocal evidence of *N*-myristoylation is currently lacking the human orthologue, or where the human orthologue does not possess an N-terminal glycine. At 72 hpf, an increased abundance of YnMyr-labelled proteins is observed in the *acbd6* crispant, including Marcks and Chchd-related proteins, known to be co-translationally *N*-myristoylated in humans (Fig. 7A). Of note, zebrafish express duplications of multiple proteins, including the aforementioned Marcks and Chchd-related proteins (Fig. 7A). Conversely, enrichment of several zebrafish proteins with N-terminal glycine in zebrafish but not found with a N-terminal glycine in humans was significantly reduced in *acbd6* crispants, including Sfpq, associated with brain development, CNS neuron axonogenesis and midbrain-hindbrain boundary initiation (Fig. 7A). Similar increases in Marcks and Chchd-related proteins are observed in the *acbd6* crispants at 120 hpf, whereas Fus and Fusl are further decreased (Fig. 7B).

We next performed a whole proteomics analysis comparing wild-type and *acbd6* crispant at 72 and 120 hpf (Supplementary Fig. 19E and F). In contrast to the significant increase of YnMyr labelling observed in *acbd6* crispants (Fig. 7A and B), the abundance of *N*-myristoylated proteins such as Marcks and Chchd-related proteins is not significantly increased at the whole proteome level at 72 and 120 hpf, further indicating the role of *acbd6* on the process of *N*-myristoylation in zebrafish. Meta-analysis of upregulated proteins in *acbd6* crispants revealed a significant involvement of translation- and metabolism-related pathways (Supplementary Fig. 20A) and significant enrichments in disease networks including frontotemporal dementia and delayed speech and language development (Supplementary Fig. 20B). Downregulated proteins are significantly involved in pathways related to nervous system development as well as neurodegeneration (Fig. 7C). Enriched disease-related networks include dystonia muscle spasticity and movement disorders, a striking similarity with the observations in affected individuals (Supplementary Fig. 20C). At 120 hpf, a meta-analysis of the whole proteome data reveals similar enrichments (Supplementary Fig. 20D–G). Notably, upregulated proteins enrich in translation- and metabolism-related pathways, whereas downregulated proteins further enrich in pathways of neurological development and disease, including spasticity (Supplementary Fig. 20G).

### Proteomics analyses of developing wild-type and *acbd6* crispant *Xenopus tropicalis*

Similar to the chemical proteomics analyses in zebrafish, we used YnMyr labelling to identify *N*-myristoylated substrates, and the pathways affected by the loss of *acbd6* in developing *Xenopus.* Here, wild-type, crispant 68 or crispant 71 *X. tropicalis* were metabolically labelled from 1 hpf to 18 hpf, due to the previously reported growth arrest. Chemical proteomics of YnMyr labelling revealed >10 *X. tropicalis* proteins the human orthologues of which are known to be co-translationally *N*-myristoylated, including a duplication of Marcks, as well as *X. tropicalis* proteins which share the N-terminal glycine with their orthologue in humans (Supplementary Fig. 21A–C). Comparing wild-type with *acbd6* crispant 68 and 71 (Fig. 7D and E) reveals a marked depletion of YnMyr-labelled proteins in the crispants, including all identified *X. tropicalis* proteins with co-translationally *N*-myristoylated human orthologues. Both crispants reveal prominent and significant reductions in proteins including Marcks, Ppm1b and Ppm1g. Whole proteome analysis revealed Marcks and Fus are markedly reduced in *acbd6* crispant 68, while Ppm1a is slightly increased, and in *acbd6* crispant 71, both Marcks and Ppm1b are reduced (Supplementary Fig. 21D and E). Meta-analysis (Supplementary Fig. 22) revealed upregulated proteins in crispants 68 and 71 are significantly affected in pathways of translation and metabolism, and notably, the ‘Parkinson-disease’-specific disease network was significantly enriched (Fig. 7F).

## Discussion

### A neurodevelopmental syndrome with progressive movement disorders characterizes *ACBD6-*related disease

Despite their predicted roles in cellular lipid metabolism, the functions of many of the ACBDs still remain unclear, as is the consequence of ACBD protein defects on human pathophysiology. In this study, we identified novel and ultra-rare bi-allelic predicted LOF variants in *ACBD6* as the disease cause in 45 previously undiagnosed individuals from 28 unrelated families. A wide age range of the cohort members (1–50 years) delineated the age-related clinical spectrum and the natural history of the *ACBD6*-related disease. The disease had an invariably early-onset and inevitably progressive course with significant motor and cognitive deterioration upon reaching adulthood, a course suggestive of underlying neurodegeneration. The phenotype is complex involving the constellation of extrapyramidal, pyramidal, and cerebellar ataxia symptoms associated with GDD/ID, microcephaly, and variable epilepsy. Impaired expressive language, delayed gait acquisition, and early-onset stooped posture with lateral trunk flexion (Pisa syndrome) were among the important pathognomonic features. Additionally, tics and tic-like vocalizations seen in six affected individuals are peculiar features associated with several neurological disorders, particularly with chorea-acanthocytosis.^35^ Whilst the present cohort of patients with *ACBD6-*related disease did have facial dysmorphism, it did not suggest a recognizable facial ‘gestalt’. The most common dysmorphic feature in the cohort was a broad chin. Of note, most of the patients had hypoplasia/agenesis of the corpus callosum and anterior commissure, suggesting a potential role for *ACBD6* in axonal pathfinding and corpus callosum development. Interestingly, claval hypertrophy observed in the present cohort is a well-documented neuroradiological sign of *PLA2G6*-associated neurodegeneration.^36^

Defects in numerous genes and pathways are known to present with the constellation of symptoms observed in *ACBD6-*related disease, particularly, with various combinations of dystonia, parkinsonism, ataxia, and spasticity.^37-40^ Their clinical phenotypes are typically classified according to the predominating symptom; however, a later approach tends to define this with a spectrum of genetic dystonia-ataxia, parkinsonism-dystonia/ataxia, and ataxia-spasticity syndromes.^37-40^ Thus, several forms of complicated hereditary spastic paraplegia, spastic ataxia, and young-onset dystonia-parkinsonism syndromes may overlap with *ACBD6* phenotypes. A suggested differential diagnosis with the disease pathways involved is given in Supplementary Table 7.

It has been suggested that neurodevelopmental abnormalities and neurodegeneration could share several molecular and cellular mechanisms. For instance, proteins, such as Aβ, MAPT/tau, Rac1, progranulin, huntingtin, PINK and parkin, frequently implicated in Alzheimer’s disease, Parkinson’s disease and Huntington’s diseases are important for nervous system development.^41^ A wide range of multisystem genetic disorders could present with a biphasic course where a complex neurological phenotype gradually evolves on the background of a pre-existing neurodevelopmental disorder.^42,43^ Therefore, we propose a likely clinical continuum associated with *ACBD6*-related disease, characterized by a combination of neurodevelopmental abnormalities and neurodegeneration.

### Zebrafish *acbd6* knockouts recapitulate many features observed in affected individuals

In recent years, the zebrafish has gained significance as an animal model for investigating neurodevelopmental disorders, owing to its high physiological similarity to humans and its responsiveness to genetic and pharmacological interventions.^44,45^ The acyl-CoA binding domain of human and zebrafish ACBD6 share significant identity and similarity in the ankyrin-repeat motif and acyl-CoA binding domain, with 80% identity and 95.4% similarity in the former and 69.2% identity and 78.2% similarity in the latter. Zebrafish models for ACBD proteins do not currently exist; however, by generating *acbd6* knockouts in zebrafish, we observed similar phenotypes to those of affected individuals, such as movement disorders, seizures, and facial dysmorphology. Our findings suggest that *acbd6* is critical for animal development, as the loss of this protein results in severe global developmental delay and increased mortality over time, as evidenced by stunted growth and severe brain development impairment by 30 dpf. Moreover, we observed motor neuron over-branching during development and progressive muscle loss, suggesting a combination of muscle and neuronal degeneration leading to movement abnormalities. Furthermore, the knockout zebrafish demonstrated increased locomotor behaviour in the dark, potentially indicating seizure or anxiety-like behaviour, similar to that seen in affected patients. In summary, the Acbd6 zebrafish models offer a promising tool for gaining deeper mechanistic insights into the role of *acbd6* and for screening potential therapeutic interventions, as zebrafish are ideal for high-throughput *in vivo* drug screening. As such, the *acbd6* model represents a valuable resource for drug discovery research.

### *Xenopus tropicalis acbd6* knockouts have gastrulation failure, brain defects and reduced locomotion

*Xenopus tropicalis* is a diploid clawed frog with a genome that contains over 80% of identified human disease genes and importantly is syntenic with over two-thirds of the human genome.^46^ Features of this animal include rapid external development and a transparent tadpole. A deep understanding of *Xenopus* biology throughout the last 70 years,^47^ combined with ease of genetic manipulation,^48^ makes it powerful for modelling disease genes^49,50^ and understanding genetic disease mechanisms.^51-54^

In the present study, the inactivation of *acbd6* in *X. tropicalis* caused severe cell movement failures during gastrulation. Marcks requires *N*-myristoylation to act in early development^55^ and is required for normal cell movement during gastrulation in frog.^56^ It is interesting to speculate that the loss of *acbd6* may cause gastrulation defects due to a loss of Marcks *N*-myristoylation; an aspect that requires future work. Owing to embryo deaths at gastrula stages, a caveat for the interpretation of the subsequent phenotype analysis is the unusually high mosaicism of these crispant tadpoles. Despite this, there were clear phenotypic differences between the control and crispant tadpoles. In some cases, the change predominated in one-half of the tadpole due to mosaicism (on the left-right axis; see second image from the left in Fig. 5G). These differences included microcephaly, reduced movement, eye abnormalities and brain structure differences, data that strengthen the link between *ACBD6* variants and the pathology observed in the patient cohort.

### *ACBD5* and *ACBD6* have different cellular localizations but both exhibit a neurodegenerative phenotype

ACBD5 is a peroxisomal membrane protein. Although current knowledge of the phenotype associated with *ACBD5* defects is limited to only four reported families,^4-7^ similar to *ACBD6*, individuals with defective *ACBD5* seem to exhibit a neurodegenerative disease, albeit with a different range of associated symptoms. Our study on peroxisome morphology and function with ACBD6 deficient and control fibroblasts did not reveal significant alterations as has been demonstrated in ACBD5 deficiency. Furthermore, Myc-ACBD6 did not localize to peroxisomes when expressed in COS-7 cells.

### *ACBD6* deficiency alters myristate probe YnMyr incorporation into substrates of NMT

Our chemical proteomics analysis comparing *N-*myristoylated proteins in fibroblasts, derived from a patient and their healthy sibling, revealed significant differences in YnMyr-labelled proteins. YnMyr incorporation was significantly higher in the ACBD6-deficient patient fibroblasts, spanning 68 known co- and 18 post-translationally *N*-myristoylated proteins. Interestingly, an apparent differential variation of YnMyr incorporation in the identified NMT substrates between healthy and patient fibroblasts was also observed. Our investigation in *ACBD6* deficient fibroblasts might indicate its role in *N*-myristoylation of a subset of NMT substrates, but further studies are required to investigate this putative role of ACBD6. Furthermore, to study the effect of ACBD6 deficiency on *N*-myristoylation, future analyses will need to focus on cell types likely directly impacting the pathways involved in e.g. neurological development.

Both the zebrafish and *X. tropicalis* model systems recapitulate many of the identified human phenotypes, including movement disorders, seizures, facial dysmorphology and developmental defects. This evidence for notable evolutionary conservation of ACBD6 function across mammals, amphibia and teleosts emphasizes the power of multiple, non-mammalian models as a method for rapid and cost-effective human gene analysis. Through our chemical proteomics analysis, coupled with whole proteome and meta-analysis, we identified that *acbd6*-deficiency in developing zebrafish embryos results in a prominent increase in metabolic labelling of known co- and post-translationally *N*-myristoylated proteins with YnMyr, suggesting *N*-myristoylation is dependent on a direct or indirect interaction between *acbd6* and the *N*-myristoyltransferases. Meta-analysis furthermore revealed that *acbd6* deficiency provokes an overall increase of proteins *N*-myristoylated with YnMyr, with a subset involved in the development of the eye, neuron and muscle, being reduced. Overall, this suggests that *ACBD6* is involved in the *N*-myristoylation of a subset of *N*-myristoyltransferase substrates that require modification and might indicate activation of a rescue mechanism in response to loss of *ACBD6*. Concomitantly, potentially compromised myristic acid-CoA binding and shuttling to *N*-myristoyltransferases in ACBD6-deficient cells may upregulate salvage pathways to increase cellular myristate concentration. For example, acyl-CoA synthases can activate exogenous lipids directly, potentially bypassing or compensating loss of ACBD6. In this case, YnMyr-CoA could more effectively compete with endogenous myristic acid-CoA for protein *N*-myristoylation than in healthy subjects, leading to the observed enhanced YnMyr labelling, suggesting exogenous myristic acid supplementation through dietary supplementation might have the potential to reduce the burden of ACBD6 deficiency. The determined differences in protein *N*-myristoylation between the skin fibroblasts derived from a single patient and unaffected sibling align with the YnMyr chemical proteomics findings from our zebrafish study. Consequently, the YnMyr chemical proteomics data of the human patient and unaffected fibroblast serves only as qualitative confirmatory evidence of the zebrafish study. For this purpose, we did not seek further replication of these findings across multiple patient cell lines.

In contrast to *ACBD6* deficient human fibroblasts and zebrafish crispants, the *acbd6 X. tropicalis* crispants show a prominent and significant reduction of YnMyr labelling, including proteins such as Marcks, Chchd, Ppm1b, Ppm1g and Fus, which are significantly involved in development. Meta-analysis indicates the upregulation of neurological and sight-related disease networks upon *acbd6* loss. The significant effect of *acbd6* loss on *X. tropicalis* development at the gastrula stage precludes a clear interpretation of the effect of *acbd6* knockout on *N*-myristoylation due to the presence of confounding changes in other processes during the period of metabolic labelling. The clearly non-viable state of the *X. tropicalis* embryos likely leads to defects in lipid metabolism, protein translation and related processes over this period which go beyond the direct impact of *acbd6* on cells.

In summary, we have shown that bi-allelic pathogenic variants in *ACBD6* are associated with a new and distinct neurodevelopmental disease with a complex and progressive dystonia-parkinsonism-ataxia phenotype. Zebrafish and *X. tropicalis* crispants recapitulate the main clinical features of the cohort with affected pathways underlying translation, metabolism, neurological development, and neurological diseases. Further studies are warranted to delineate the clinical phenotype and understand the pathomorphological presentation and molecular mechanisms of the *ACBD6*-related disease. This includes elucidating the molecular interplay between ACBD6 and *N*-myristoyltransferases involved in the co- and post-translational modification of nascent protein chains, how ACBD6 affects lipid metabolism and the identification of treatments of ACBD6 deficiency.

## Supplementary Material

awad380_Supplementary_Data

## Data Availability

The data that support the findings of this study are available from the corresponding author, upon reasonable request. The mass spectrometry proteomics data have been deposited to the ProteomeXchange Consortium via the PRIDE^57^ partner repository with the dataset identifiers PXD024957 (YnMyr chemical proteomics in human cells), PXD043676 (YnMyr chemical proteomics in zebrafish), PXD043679 (zebrafish whole proteome), PXD043677 (YnMyr chemical proteomics in *X. tropicalis*) and PXD043680 (*X. tropicalis* whole proteome).
